# Liking Product Landscape: Going Deeper into Understanding Consumers’ Hedonic Evaluations

**DOI:** 10.3390/foods8100461

**Published:** 2019-10-09

**Authors:** Claudia N. Sánchez, Julieta Domínguez-Soberanes, Héctor B. Escalona-Buendía, Mario Graff, Sebastián Gutiérrez, Gabriela Sánchez

**Affiliations:** 1 Facultad de Ingeniería, Universidad Panamericana, Aguascalientes 20290, Mexico; cnsanchez@up.edu.mx (C.N.S.); jsgutierrez@up.edu.mx (S.G.); 2 CONACYT—INFOTEC Centro de Investigación e Innovación en Tecnologías de la Información y Comunicación, Aguascalientes 20313, Mexico; 3 Escuela de Negocios Gastronómicos, Universidad Panamericana, Aguascalientes 20290, Mexico; 0171350@up.edu.mx; 4 Departamento de Biotecnología, Universidad Autónoma Metropolitana Iztapalapa, Ciudad de Mexico 09340, Mexico; hbeb@xanum.uam.mx

**Keywords:** consumers’ perceptions, consumers’ preferences, data analysis, liking product landscape, market segmentation, sensory analysis

## Abstract

The use of graphical mapping for understanding the comparison of products based on consumers’ perceptions is beneficial and easy to interpret. Internal preference mapping (IPM) and landscape segmentation analysis (LSA) have successfully been used for this propose. However, including all the consumers’ evaluations in one map, with products’ overall liking and attributes’ perceptions, is complicated; because data is in a high dimensional space some information can be lost. To provide as much information as possible, we propose the liking product landscape (LPL) methodology where several maps are used for representing the consumers’ distribution and evaluations. LPL shows the consumers’ distribution, like LSA, and also it superimposes the consumers’ evaluations. However, instead of superimposing the average overall liking in one map, this methodology uses different maps for each consumer’s evaluation. Two experiments were performed where LPL was used for understanding the consumers’ perceptions and compared with classic methodologies, IPM and cluster analysis, in order to validate the results. LPL can be successfully used for identifying consumers’ segments, consumers’ preferences, recognizing perception of product attributes by consumers’ segments and identifying the attributes that need to be optimized.

## 1. Introduction

Sensory analysis techniques are essential to establish the quality of products and to understand consumer preferences [1]. Traditionally, there are two ways of performing sensory analysis of products. The first one is selecting and training a panel of assessors who after a period become experts in the evaluation of a specific type of product. This is useful for performing a comparison among products based on their attributes [2,3] or for guaranteeing the quality and sensory properties of a product [4]. This research is based only on consumers’ evaluations that grade the products. Based on the ISO 11136–2014, a consumer is restricted to a person who is not an expert, an expert sensory assessor or a selected assessor. Understanding the consumers’ perceptions of products, based on their attributes, is the realm of sensory analysis because the success of a product depends on the consumers, taking into consideration the ones that consume the food product and make the purchase decision [5]. In addition, Worch et al. [6] performed a study comparing expert and consumers’ profiles for 12 perfumes. They concluded that no significant differences exist between the two panels that analyzed the products. Rothman and Parker affirmed in [7] that it is very common to combine hedonic judgments, preference or acceptance measures, with just-about-right (JAR) scales to understand consumers’ perceptions and to provide information concerning which product attributes to adjust and in which direction, which means to increase or decrease each attribute when making a reformulation. We used a hedonic scale of nine points for representing the overall liking of products and a JAR scale of five points for understanding the perception of products’ attributes. The information obtained by JAR provides an idea on how to optimize a prototype [7,8,9,10,11]. The simplest way to analyze JAR data across products and attributes is the graphical method, where a bar graph allows visualizing the frequency distribution of the scale values [12]. Another well-known technique for analyzing JAR evaluations is the Thurstonian ideal point modeling [13] where the probabilistic distribution of the product attribute is compared against the probabilistic distribution of the ideal. However, those techniques analyze JAR responses as a whole, making it necessary to understand how the perception of attributes affects the products’ overall liking. Penalty analysis or mean drop analysis [14] is a method for determining whether consumers who do not find a particular attribute JAR can rate the product lower on the overall liking, when compared to those who find the same attribute JAR. Some other techniques that analyze the influence of attributes’ perception on overall liking are [11,15,16,17,18]. Horn and Ford described in [19] how a multivariate graphical could be used for representing the products in a multidimensional space defined by the attributes measured using a JAR scale. In this case, the correspondence analysis or principal component analysis can be used.

Preference mapping is of use primarily in research and food development. It can establish: (1) The most liked products, (2) patterns of consumer liking through segmentation based on demographic and socio-cultural differences, (3) reasons for liking and rejecting products and (4) the drivers of liking. Preference mapping can give a clear idea of what changes must be made in product reformulation [20,21,22,23,24]. Preference mapping is divided into two categories: Internal preference mapping (IPM) and external preference mapping (EPM). IPM and EPM are complementary. The first one is useful for understanding the dominant products based on the analysis of consumers’ overall liking [25]. The latter allows the comparison of products based on their attributes or physicochemical characteristics [26].

Preference mapping applies multivariate statistical techniques that have evolved to provide a better understanding of the data [27]. Multidimensional preference mapping (MDPREF) was the first IPM based on multidimensional scaling. Nonetheless, it later adopted the principal component analysis (PCA). In IPM, the consumers’ hedonic evaluations of the products are used to create a multidimensional space where two principal components are used to visualize the map [28]. Internal preference mapping creates a preference map where consumers are represented with vectors; the direction indicates which products are preferred and the vectors’ length represents the grade of likeness. Some of the studies that have used IPM for understanding consumers’ preferences are [25,29,30,31,32]. External preference mapping (EPM), as IPM, uses PCA, but the attributes or physicochemical characteristics of products define the multidimensional space where the products are placed [26]. Several variants of IPM and EPM maps have been developed. Danzart proposed different models [33,34] using quadratic and elliptic functions that allow superimposing consumers’ preferences in EPM. Danzart´s proposal used the average of consumers’ overall liking scores across products for each individual. However, averaging adds much noise to the map. Besides, documents that used Danzart’s methodology [27,35] affirmed that elliptical and quadratic models are seldom used in practice because they are often difficult to interpret. Delarue et al. [36] divided the sensory preference map into two: Preference or rejection of the product. They considered that the preference or rejection zone could be less arbitrary. Faber et al. [35] reduced the error of the models that superimpose the consumers’ preferences in EPM by increasing the number of principal components used. Le et al. proposed consumers’ preference analysis [37] based on IPM where the physico-chemical or descriptive attributes of products are superimposed and different maps, one for each attribute, are compared. Multidimensional scaling (MDS) was used to create an EPM based on consumers’ words and hedonic evaluations [38]. Næs et al. used the preference mapping method combining PCA and ANOVA to understand conjoint data that included consumer information and liking. The analysis revealed that PCA and ANOVA with a non-specialized system of analysis provide a clear idea on how conjoint data interact, even when two variables are of different types: Continuous and categorical [21]. PrefMFA, proposed in [39], combines internal and external preference mapping techniques, it is based on multiple factor analysis that defines the dimensional space based on both product properties and hedonic consumers’ scores. In the vector space, two products are close if they have similar sensory profiles, meaning that they are either liked or disliked. They used the quadratic model proposed by Danzart to superimpose the liking scores in the map. Landscape segmentation analysis (LSA) [40,41,42] is widely used in practice for its easy interpretation. It is based on ideal point behavior of consumers. In this map, dark contours are representative of a liking segment. Products close to dark contours indicate the existence of a dense consumers’ group who like the product. Other maps are based on the ideal profile methodology [43,44,45,46].

The objective of this manuscript is to propose a visualization method, called liking product landscape (LPL), which is capable of identifying consumers’ segments and consumers’ preferences, recognizing the perception of product attributes and identifying that attributes that need to be optimized. It helps to make decisions when a new product is being launched into the market, to compare products and to analyze whether a product needs to be reformulated. It is based on consumers’ evaluations using a hedonic scale for overall liking and a JAR scale for attributes. Consumers are positioned in a multidimensional space and several techniques for mapping that space to a plane are tested, such as principal component analysis and multidimensional scaling. The consumers’ distribution is represented, as landscape segmentation analysis [40,41,42], to identify consumers’ segments. Besides, the consumers’ evaluations are superimposed with a regression model, similar to Danzart’s proposal [33,34]. We created several maps, one for each evaluation, as consumers’ preference analysis (CPA) [37], intending to reduce the noise when averaging grades. Support vector machines were used for calculating the regression model and then compared with the original model proposed by Danzart. The difference between LPL and CPA is that CPA only uses different colors when plotting the points, whereas LPL uses a regression model; in addition, it incorporates a consumers’ distribution map. Besides, as penalty analysis, LPL allows analyzing whether consumers who do not find a particular attribute JAR rate the product lower on the overall liking than those who find the same attribute JAR. However, in LPL we use a visualization technique. Moreover, we released our LPL Python implementation as open-source [47].

## 2. Materials and Methods

### 2.1. Materials

The data is related to red wine, where the interest is to understand the overall liking and the perception of five attributes: Sweetness, acidity, astringency, body and fruitiness. Five different wines were used for the sensory tasting. The wines were chosen with different sensory characteristics: Wine 1: Dry, dense and astringent (Crápula, Mucia Spain, 2013); wine 2: Sweet, acid and fruity (Viña Zorzal, Garnacha, Navarra Spain, 2015); wine 3: Sweet and dense (Lloro de Tierra, Mistela, Dolores Hidalgo, Guanajuato México, 2014); wine 4: fruity, dry and light (Paco García, Rioja Spain, 2015); wine 5: Fruity, sweet and dense (Semillón Gewurztrainer, Valle de Colchagua, Chile 2008). Two experiments were used for showing how our methodology complements the use of IPM for understanding consumers’ evaluations. Table 1 presents information about experiments.

**Experiment 1:** The characteristics of the five wines were assumed as follows. Wine 1: dry, dense and astringent. Wine 2: Sweet, acid and fruity. Wine 3: Sweet and dense. Wine 4: fruity, dry and light. Finally, wine 5: fruity, sweet and dense. Two consumers’ segments were designed thinking of women and men profiles (see Table 2). The assumed evaluations are the overall liking that corresponds to a hedonic scale of nine points and to JAR for five wine attributes: Sweetness, acidity, astringency, body and fruitiness. These were evaluated using a five-point JAR scale: Where 0 represents just about right, −2 too low quantity and +2 too much. One hundred consumer evaluations were simulated, 50 women and 50 men, adding Gaussian noise to the values presented in Table 2.

**Experiment 2:** Students (*n* = 100) from the Universidad Panamericana, Aguascalientes, México, were invited for sampling wine, their age ranged 22–25 years. They were divided into male (*n* = 50) and female (*n* = 50). Subjects were instructed not to eat or drink anything two hours before the experiment. They were asked to taste a sample of 30 mL of each wine. After sampling each wine, consumers were asked to drink water and to eat a piece of cracker, so the aftertaste of each wine would not interfere in the analysis. After sampling each wine, they were asked to complete a sensory questionnaire, in which a just about right (JAR) scale of five points was used for evaluating the attributes: Sweetness, acidity, astringency, body and fruitiness. Besides, a hedonic scale of nine points was used for grading the overall liking of the product. As it is recommended in [48], we pretested the questionnaire to confirm that it is understandable and that subjects would be able to follow the instructions for the test. Moreover, based on ISO 11136–2014, the samples were anonymously presented to consumers using a three-digit random code.

### 2.2. Methods

**Figure 1:**
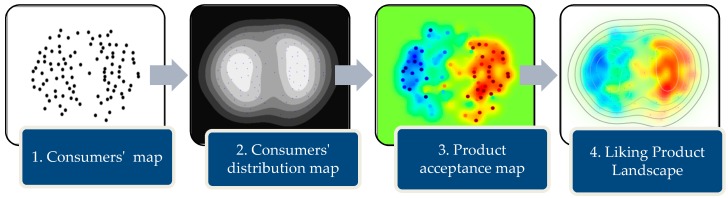
Liking product landscape creation process.

The process of creating the liking product landscape (LPL) is divided into four steps (see Figure 1). The first step is to calculate the consumers’ map, where all the consumers are positioned in a map. The idea is plotting together consumers who gave similar evaluations, whereas consumers who evaluated the products differently are plotted apart. Then, a consumers’ distribution map is used to represent the distribution of consumers and identify market segments. A product acceptance map superimposes in the consumers’ map the evaluations of a product’s overall liking or an attribute evaluation of a specific product for analyzing the consumers’ perceptions. ’Superimposes’ is when a regression model is used for creating a landscape of the consumers’ evaluations (see the product acceptance map in Figure 1). Finally, liking product landscape (LPL) mixes in one map the consumers’ distribution map and the product acceptance map that allows analyzing the consumers’ segments and the grading. All the implementations were done in python [49] using the libraries scikit-learn [50] and pandas [51].

**Step 1.** Consumers’ map: The inputs of the methodology are the numerical evaluations done by consumers. For example, let us assume the evaluation of four products based on overall liking plus adding sweetness and acidity as perception attributes. These conditions provide twelve values. Consequently, the consumers can be represented as vectors, i.e., the vector of the consumer *i* is $x_{i}\;\in \;{\mathbb{R}}^{d}$, where *d* represents the number of evaluations per consumer. In the majority of the cases, *d* > 2, so it is impossible to plot those consumers’ vectors in a plane. For this reason, a dimensional reduction technique that can map the consumers’ vectors $x_{i}\;\in \;{\mathbb{R}}^{d}$, to $x_{i}^{*}\;\in \;{\mathbb{R}}^{2}$, is needed. Therefore, we compared the use of two well-known reduction techniques: Principal component analysis (PCA) and multidimensional scaling (MDS).

Principal component analysis (PCA) is an orthogonal projection of the data onto a lower-dimensional space such that the variance of the projected data is maximized [52,53]. This method has been widely used in sensory analysis [54,55,56,57,58,59,60,61]. Furthermore, it is a core component of the internal and external preference mappings. In IPM, the consumers define the space and the products are vectors whose entries are the grades of each consumer. On the other hand, in EPM, the products are points in a space defined by their physicochemical attributes [2]. In both cases, PCA is used to reduce the dimensionality of the vector space and to visualize the products in a plane. We used PCA to reduce the dimensionality of consumers’ vectors, $x_{i}\;\in \;{\mathbb{R}}^{d}$, and map them into a two-dimensional space, i.e., $x_{i}^{*}\;\in \;{\mathbb{R}}^{2}$. We used two approaches; the first one consisted in performing a classic internal preference map (IPM) fed only with the overall liking of all products given by consumers. The new consumers’ vectors are calculated using only the first two principal components and multiplying the factor loadings by the variance of the product points in the new space. The first two principal components are those that explain the highest amount of variance. The second approach is performing a traditional PCA; it could be fed with the data of overall liking (OL), the sensory perceptions (JAR) or both of them (OLJAR).

Multidimensional scaling (MDS), described in [62], is a technique that, given the distances among consumers’ vectors in the original space, $x_{i}\;\in \;{\mathbb{R}}^{d}$, computes the new consumers’ vectors, $x_{i}^{*}\;\in \;{\mathbb{R}}^{2}$, trying to preserve those distances. In this research, the distances among consumers’ vectors are the Euclidean distances. MDS solves an optimization problem that minimizes:

(1) $$stress=\sqrt{\sum_{i<j}\left(d_{ij}-\hat{d_{ij}^{\;}}\right)\;/-\sum_{i<j}d_{ij}^{2}\;},$$

where $d_{ij}$ and $\hat{d_{ij}^{\;}}$ are the distances between the i-th and j-th consumers’ vectors in the original ${\mathbb{R}}^{d}$ and new ${\mathbb{R}}^{2}\;$ spaces. It allows visualizing the consumers based on their evaluations and groups of consumers’ points in the generated map indicate that the evaluations of that group are very similar. Cartier et al. [63] also used MDS to find optimal consumers’ positions based on consumers’ evaluations. As well as PCA, it could be fed with the data of overall liking (OL), the sensory perceptions (JAR) or both of them (OLJAR).

**Step 2.** Consumers’ distribution map: Once all the consumers’ vectors have been positioned in a two-dimensional space, kernel density estimation (KDE) with Gaussian kernel is used to visualize the consumers’ distribution (see Figure 1). KDE is a technique that calculates the smoothness estimation of a density function. It was introduced by Rosenblatt [64] and explained in detail in [52] and [65]. The consumers’ density p(x) is calculated by a sum of Gaussian functions centered on all the consumers’ points. Given n consumers $x_{1}^{*},\;x_{2}^{*},\;\ldots ,\;x_{n}^{*}$ in a 2-dimensional space, the kernel density estimator is defined as:

(2) $$p\left(x\right)=\left(\frac{1}{2}\pi nh^{2}\right)\underset{i}{\sum }\exp\left(-\frac{\|x-x_{i}^{*}\|{}^{2}}{2h}\right)$$

where *x* represents one point in the consumers’ map, *n* is the number of consumers, ‖.‖ represents the vector norm and h  represents a bandwidth to control the smoothness of the estimated densities. *h* is calculated using the Scott rule [66], that is:

(3) $$h=n^{-1/6}$$

**Step 3.** Product acceptance map: The goal of the product acceptance map is to understand consumers’ evaluations, which is one of the main objectives of sensory analysis. It is based on Danzart’s proposal [33,34]. Given the consumers’ vectors and consumers’ grades, a regression model that represents liking is created. Danzart’s original proposal is to use the average of the overall liking across products for each consumer. However, it has been noted by [36] that the average reduces the level of information and could be conducive to an erroneous conclusion. For this reason, our methodology creates one map per evaluation intending to minimize the error.

We propose two techniques for calculating the landscape that models the consumers’ evaluations: A quadratic model, as the Danzart’s proposal and support vector machines. The main idea is as follows. Given n consumers’ vectors $x_{1}^{*},\;x_{2}^{*},\;\ldots ,\;x_{n}^{*},\;x_{i}^{*}\in {\mathbb{R}}^{2}$ and n consumers’ grades $g_{1},g_{2},\;\ldots ,\;g_{n},\;g_{i}\in \mathbb{R}.$ The objective is to find the function f that minimizes the difference between $\;f\left(x_{i}^{*}\right)$ and $g_{i}$. The Quadratic Model (QUA) is defined as:

(4) $$f\left(x_{i}^{*}\right)=\alpha_{0}+\alpha_{1}d_{1}+\alpha_{2}d_{2}+\alpha_{3}d_{1}^{2}+\;\alpha_{4}d_{2}^{2}+\;\alpha_{5}d_{1}d_{2}$$

where *d*_1_ and *d*_2_ represent the consumer coordinates in the consumers’ map and the α’s are calculated using ordinary least squares. On the other hand, we propose the use of support vector machines (SVM) with Gaussian kernel that have been successfully used to solve a variety of problems. Smola and Scholkopf [67] describe how SVM can be used specifically for regression problems. In this case, the inputs of the SVM are the consumers’ vectors in two-dimensional space and the outputs are the grades. Figure 1 shows an example of a product acceptance map constructed with SVM, the points and their colors represent the consumers’ and the grade values, respectively, red spots are consumers that give higher grades and in the opposite, blue points are consumers that give lower grades.

Most regression models have small errors. Figure 1 shows a product acceptance map where the consumers’ points are represented with the color of their evaluations. It can be seen that the landscape color represents the general tendency of consumers’ evaluations, but there is an error. To measure the effectiveness of the models that describe the consumers’ evaluation, we use the mean absolute error (MAE), which is defined as:

(5) $$MAE=\frac{1}{n}\underset{i}{\sum }\left|f\left(x_{i}^{*}\right)-g_{i}\right|$$

To facilitate the understanding, the MAE is multiplied by 100 and divided by the number of points in the evaluation scale to represent the error in a range between 0 and 100%. We called it ’percentage error’. For example, the percentage error in the product acceptance map that appears in Figure 1 is 18.1%.

**Step 4.** Liking product landscape: Finally, liking product landscape (LPL) is the combination of the consumers’ distribution map and the product acceptance map. Figure 1 shows this combination. Transparency is added to represent the consumers’ distribution; this means areas without consumers are plotted totally in white; in contrast, areas with brilliant colors represent dense consumers’ zones. Besides, a contour plot is used to represent the density of consumers.

**Figure 2:**
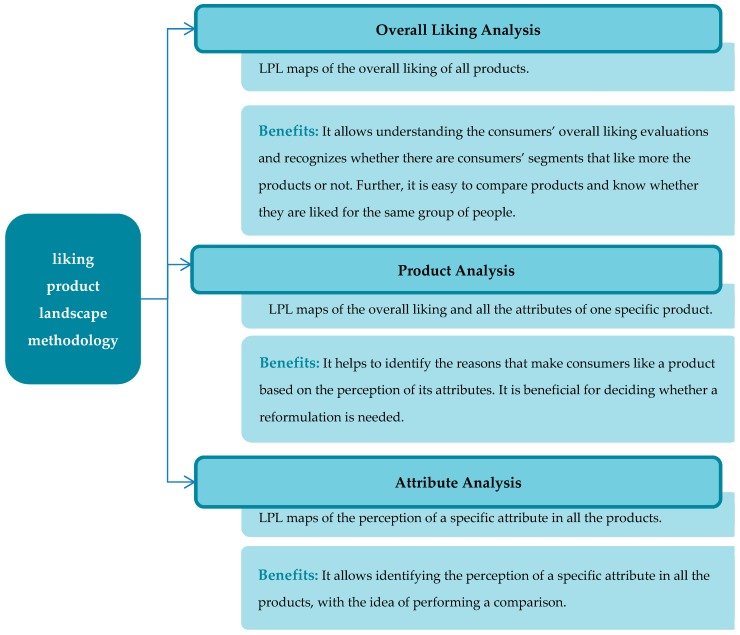
Analysis that can be performed using the liking product landscape methodology.

The LPL maps are grouped into three different analyses (see Figure 2): Overall liking analysis (see examples in the Figure 5 and Figure 8), product analysis (see an example in Figure 9) and attribute analysis (see an example in Figure 10).

## 3. Results and Discussion

In this section, the analysis of the consumers’ evaluations of the experiments is described. Comparison between classic techniques and our methodology is performed to understand the knowledge elicited based on different approaches. In addition, we compared the use of different techniques and data for creating the LPL maps. In total, we analyzed the performance of fourteen different methodologies obtained by combining the different techniques for constructing the product acceptance map (QUA and SVM), for calculating the consumers’ map (IPM, PCA and MDS) and by combining the data that these techniques can use (OL, JAR or OLJAR).

To simplify the reading, some key colors are used. In the liking product landscape maps where the overall liking is analyzed (Figures 5, 8 and 9) red zones represent consumers who evaluated the product higher, whereas blue areas represent consumers that graded it lower. For attribute perception (Figures 9 and 10) green indicates that the attribute is JAR, yellow represents that it has more and red too much of the attribute. In contrast, blue represents a little less and dark blue too weak intensity of the attribute. A bar was added whose parts are of proportional length to the percentage of consumers that affirm that the product has a specific JAR value. Areas without consumers are plotted totally in white; in contrast, areas with brilliant colors represent dense consumers’ zones; moreover, a contour plot is used to represent the density of consumers. Some numeric values are printed, as the percentage error of the product acceptance map, the average overall liking or the JAR percentage.

In the repository [67], we released our LPL Python implementation as open source, in addition to the data and results of both experiments.

### 3.1. Results of Experiment 1

**Figure 3:**
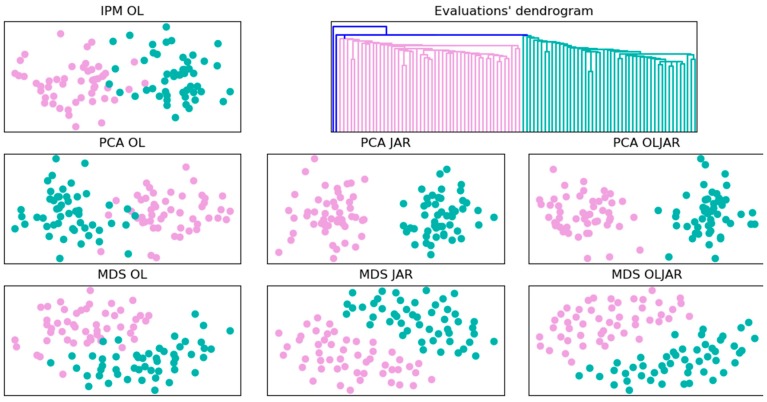
Experiment 1. Consumers’ groups, pink and green points represent women and men consumers, respectively.

The first experiment was designed to have two consumers’ segments, which are women and men. So, we expected to find these segments in the LPL maps. Figure 3 shows a dendrogram that represents the groups of consumers using the evaluation data. It corresponds to a hierarchical clustering based on Euclidian distances with Ward’s minimum variance criterion. As it was expected, two groups were found. Besides, different consumers’ maps were created using the techniques IPM, PCA and MDS (see Figure 3). The consumers’ maps created with the combination of technique data, PCA-JAR, PCA-OLJAR, MDS-JAR and MDS-OLJAR, can identify the two consumers’ segments better than the classic technique IPM. This can be explained because IPM is constructed only with the overall liking, whereas PCA and MDS can be fed with all the evaluations.

**Figure 4:**
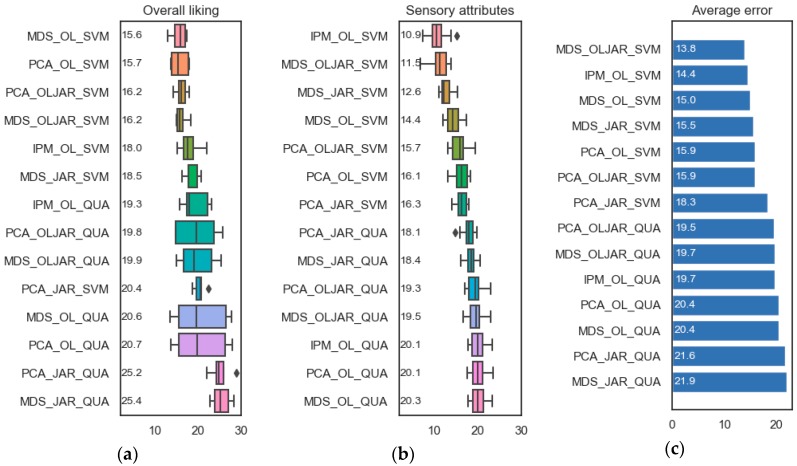
Comparison among all techniques and data that can be applied for constructing liking product landscape (LPL) maps considering the percentage error. (**a**) LPL maps’ percentage errors that represent overall liking evaluations, (**b**) LPL maps’ percentage errors that represent just-about-right (JAR) evaluations and (**c**) average errors of both evaluations. For improving the reading, the techniques are sorted based on their average percentage error, which is also shown in the figure. MDS, Multidimensional scaling; OL, overall liking; SVM, support vector machines; PCA, principal component analysis; JAR, just-about-right; IPM, Internal preference mapping; QUA, Quadratic Model.

A comparison among all techniques and data that can be applied for constructing LPL maps considering the percentage error was performed. For the creation of the consumers’ map, IPM, PCA and MDS can be applied. IPM uses the overall liking (OL) evaluations and, for PCA and MDS, different data can be used: OL, the JAR sensory perceptions or both of them OLJAR. For calculating the product acceptance map, the regression techniques QUA and SVM can be used. To facilitate reading the following notation is used: The technique for constructing the consumers’ map is followed by the symbol “_” and then comes the abbreviation of the data used for calculating that map, then another symbol “_”, which is followed by the technique for calculating the product acceptance map. Figure 4 shows the results in terms of percentage error of the LPL maps created by a combination of different techniques and data. In general, SVM produces smaller errors than QUA. It could be explained because SVM generates a more complex regression model that fits better with the data. The combination of techniques that generates the smallest average percentage error on both kind of evaluations, overall liking and sensory perceptions, is MDS_OLJAR_SVM, followed by IPM_OL_SVM. For that reason, we recommend using MDS fed with OLJAR for the construction of the consumers’ map and SVM as a regression technique in the product acceptance map. The rest of the manuscript uses this combination of techniques and data.

**Figure 5:**
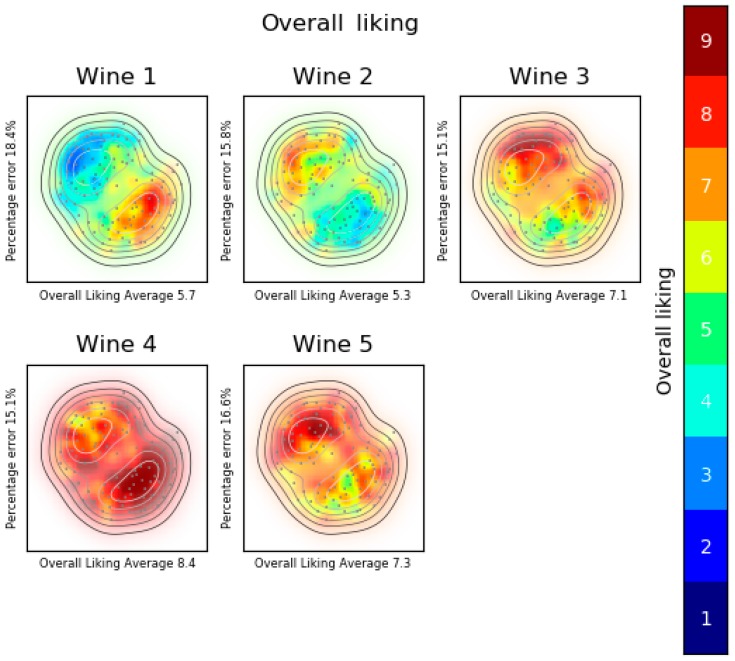
Overall liking analysis. Liking product landscape, consumers’ map created with multidimensional scaling (MDS) fed with overall liking just about right (OLJAR) and product acceptance maps created with support vector machines (SVM). The two groups correspond to women and men. Women consumers are on the top-left side and men consumers on the bottom-right side.

Figure 5 shows the overall liking analysis of the first experiment. The two groups correspond to women and men. Women consumers are on the top-left side and men consumers on the bottom-right side. Based on experimental data represented by colors, it is easy to observe that the preferred wine is wine 4 and also it can be detected that men like this wine more than women. Wine 1 is a specific case and it can be seen that a consumer segment likes it a lot (men) and the other segment dislikes it (women).

### 3.2. Results of Experiment 2

**Figure 6:**
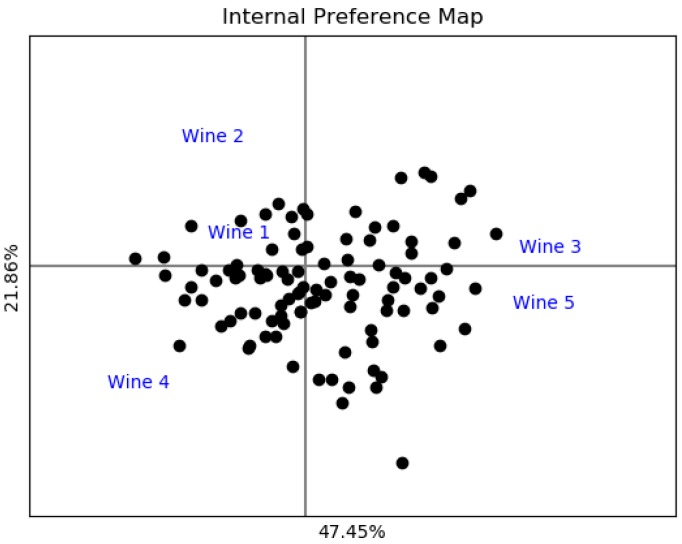
Internal preference mapping of experiment 2.

The goal of this experiment is to perform a real analysis of consumers’ evaluations. The averages of overall liking are 5.2, 4.8, 5.5, 5.5 and 5.8 for wines 1, 2, 3, 4 and 5, respectively. Figure 6 shows the IPM; any liking tendency can be observed and this is expected because the averages are similar for all the wines.

**Figure 7:**
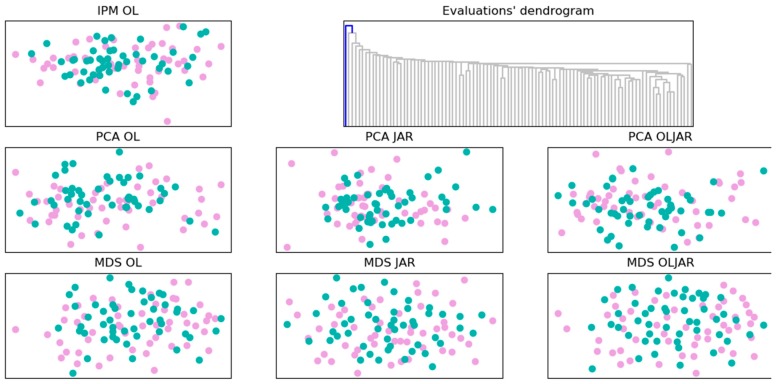
Experiment 2. Consumers’ segments; pink and green points represent women and men consumers, respectively.

Figure 7 shows the analysis of consumers by gender, similar to Figure 3. However, in this experiment, we cannot find evidence that women and men evaluate differently.

**Figure 8:**
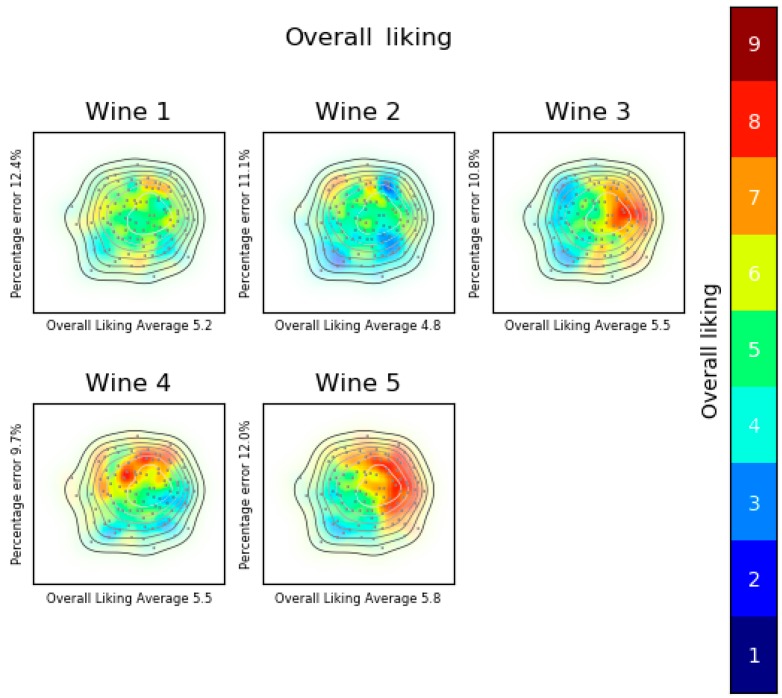
Overall liking analysis. Liking product landscape, consumers’ map created with MDS fed with OLJAR (overall liking (OL), the sensory perceptions (JAR) or both of them (OLJAR)) and product acceptance map created with SVM. Wine 1: Dry, dense and astringent; wine 2: Sweet, acid and fruity; wine 3: Sweet and dense; wine 4: Fruity, dry and light; and wine 5: Fruity, sweet and dense.

Figure 8 shows the overall liking analysis of experiment 2. The same pattern can be observed for wines 3 and 5, so they were evaluated similarly. Moreover, in the IPM (Figure 6), it can be seen that wines 3 and 5 appear together. Based on the colors, it is inferred that some consumers liked these wines a lot and others disliked them. Moreover, it can be observed that although the average overall liking is low, there is a group of consumers who evaluated highly these wines. LPL represents the overall liking in detail for each product, allowing us to recognize whether there are consumers’ segments who liked the product more or not. Consequently, it is easier to compare products and know if there are consumers’ segments that liked the same products, or some others who liked one product more than another.

**Figure 9:**
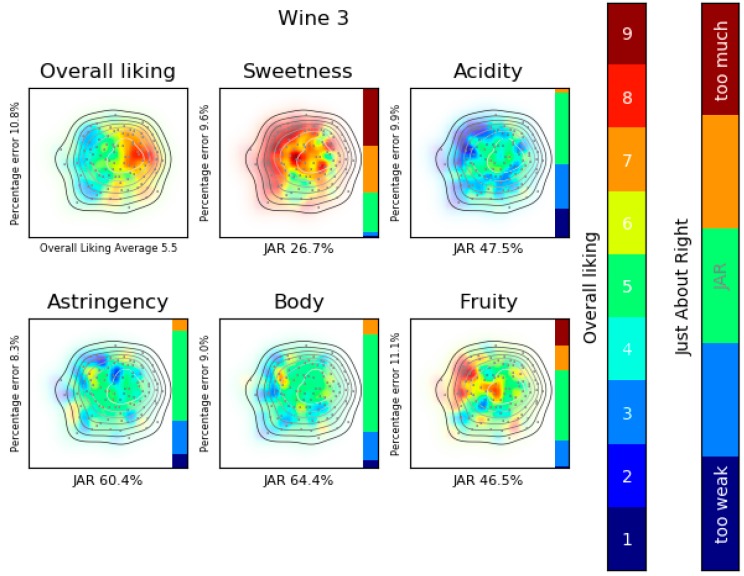
Analysis: Wine 3. Liking product landscape, consumers’ map created with MDS fed with OLJAR and product acceptance map created with SVM.

Figure 9 shows the product analysis for wine 3. Knowing that in LPL the position of consumers is the same in all the maps, it can be observed that consumers who disliked the wine also find it too sweet and too weak in acidity.

**Figure 10:**
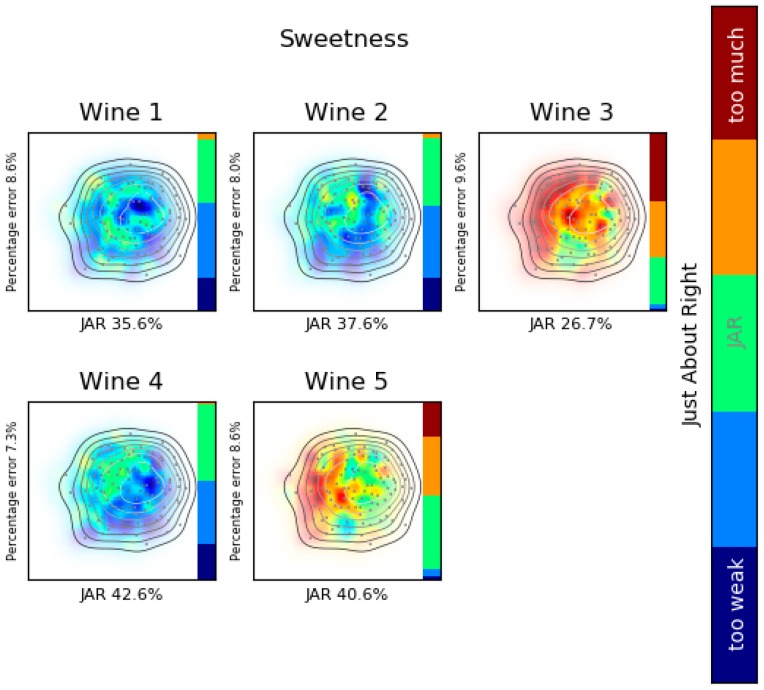
Attribute analysis: Sweetness. Liking product landscape, consumers’ map created with MDS fed with OLJAR and product acceptance map created with SVM.

Another way of presenting LPL is by attributes. Figure 10 presents the attribute analysis for sweetness. It can be seen that wine 3 is the sweetest and it is too sweet based on consumers’ perceptions. On the other hand wines 1, 2 and 4 were found too weak. This can be found by analyzing the JAR scale by itself. However, LPL shows that consumers who found wine 3 too sweet are the same who said that wines 2 and 4 are JAR sweet.

## 4. Conclusions

Liking product landscape (LPL) is a visualization mapping that allows performing an in-depth analysis of consumers’ evaluations. It can be used for understanding the overall liking and JAR scales.

Three analyses can be successfully performed. The first one is overall liking analysis, with the LPL maps of the overall liking of all products. It represents the overall liking in detail for each product allowing to recognize whether there are consumers’ segments which like the products more or not. LPL facilitates the comparison among products and it is easy to know whether they are liked for the same group of people. The second is product analysis, where the LPL maps present the overall liking and all the attributes of one specific product. This analysis helps to identify the characteristics that make a product likeable based on the perception of its attributes. It is beneficial for deciding whether a reformulation is needed. Finally, attribute analysis allows identifying the perception of a specific attribute in all the products, with the idea of performing a comparison. All LPL maps include the consumers’ distribution that helps to understand dense zones of consumers. LPL is a methodology that allows visualizing the information, not only of the overall liking but also the attributes, making it an approachable and straightforward approach to identify consumers’ perceptions by consumers’ segments.

Different techniques can be used for the construction of the LPL maps. On the one hand, PCA or MDS are possible techniques to create the consumers’ map. On the other hand, either PCA or MDS could use OL, JAR or OLJAR for representing the consumers. Moreover, QUA or SVM can be selected for fitting the regression model. In total, fourteen combinations of all techniques were compared using the percentage error based on the MAE of the acceptance maps. Based on the results, the best combination is using MDS fed with OLJAR for the construction of the consumers’ map and SVM as a regression technique in the product acceptance map, followed by IPM fed with OL for the creation of the consumers’ map and the same procedure (SVM) for the product acceptance map.

Comparing LPL with other visualization techniques, we can say that a limitation of our methodology, where PCA or MDS are used to create the consumers’ map, is that products’ points cannot be represented in the map, in contrast to IPM and LSA where products can be located. However, this is not a problem because LPL uses several maps, each one for evaluation and it allows comparison among products, specifically on the overall liking analysis and the attribute analysis, focusing on the perception of consumer segments. LPL, like LSA, includes a consumers’ distribution map to identify market segments. Similar to the Danzart’s proposal, LPL superimposes in a map the consumers’ evaluations. Based on the results, the use of SVM with Gaussian kernel instead of the Danzart’s model is recommended for reducing the error in the visualization. A strength of LPL is that in all maps the consumers are in the same location, and this allows analyzing which consumers like or dislike the products and how those specific consumers perceive the attributes, such as sweetness or astringency. It can be useful in the process of answering questions like: Why do consumers who like the product like it? and on the other hand, Why do consumers who do not like the product not like it?

## Figures and Tables

**Table 1:** Experiments.

Experiment Number	Products	Description	Number of Products	Number of Consumers
1	Red wines	A simulated experiment designed to have two consumers’ segments, women and men, that evaluate the products differently.	5	100
2	Red wines	A real experiment where consumers tested and evaluated different wines.	5	100

**Table 2:** Simulated average rating for experiment 1.

	Wine 1	Wine 2	Wine 3	Wine 4	Wine 5
**Women**					
Overall liking	3	7	8	7	9
Sweetness	−2	+1	0	−1	+1
Acidity	−1	+1	0	+1	0
Astringency	+2	0	+1	−1	0
Body	+1	0	+2	−2	+1
Fruity	−1	+1	−1	0	+1
**Men**					
Overall liking	8	4	6	9	6
Sweetness	0	+2	+2	0	+1
Acidity	0	0	0	+1	−1
Astringency	+1	−1	−1	−2	−1
Body	0	−1	−1	−2	0
Fruity	0	+2	+2	−1	0
